# Liposomal encapsulation of a synthetic bromophenol for antitumor efficacy and apoptotic activity in cancer cells

**DOI:** 10.1007/s00210-026-05094-2

**Published:** 2026-02-13

**Authors:** Bercem Dilan Oztanrikulu, Ekrem Ozdemir, Bahri Avci, Süleyman Göksu, Handan Uguz Bayrakceken, Hakan Askin

**Affiliations:** 1 Department of Molecular Biology and Genetics, Ataturk University, Erzurum, Turkey; 2 Department of Medical Services and Techniques, Ardahan University, Ardahan, Turkey; 3 Department of Chemical Engineering, Izmir Institute of Technology, Izmir, Turkey; 4 Department of Chemistry, Atatürk University, Erzurum, Turkey; 5 Department of Field Crops, Atatürk University, Erzurum, Turkey

**Keywords:** Liposomal bromophenol, Drug delivery system, Cancer therapy, Cytotoxicity, qRT-PCR

## Abstract

A novel synthetic bromophenol (BP), inspired by marine-derived natural bromophenols, was evaluated for its antitumor activity and for the enhancement of its in vitro performance through liposomal encapsulation (LipoBP). Etoposide was used as a reference in characterization, release, and loading studies. PEGylated liposomes were employed to improve BP’s solubility, bioavailability, and therapeutic potential. The cytotoxicity, apoptosis, and gene expression effects of free BP and LipoBP were assessed in A549 (lung) and MCF-7 (breast) cancer cell lines. WST-8 assays showed that encapsulation significantly increased BP’s cytotoxic activity, particularly in A549 cells, while flow cytometry and Annexin V-FITC/PI analyses indicated more pronounced apoptotic induction by LipoBP compared with free BP. qRT-PCR analyses revealed upregulation of proapoptotic genes (*BAX, CASP6, CASP3* and *CASP9*) and downregulation of antiapoptotic/survival genes (BCL-XL, IQSEC2) in both cell lines, indicating activation of intrinsic apoptotic pathways. Plain liposomes exhibited minimal cytotoxicity, confirming their biocompatibility. Liposomal bromophenol, which we have introduced to the literature for the first time, is expected to be a promising nanocarrier system that could be effective in cancer treatment by improving the therapeutic index of new drug candidates such as marine bromophenols.

## Introduction

Cancer is a heterogeneous and multifactorial disease characterized by genetic abnormalities, dysregulated signaling pathways, and uncontrolled cellular proliferation resulting from defects in cell-cycle checkpoints and apoptosis regulation (Hanahan 2022). It remains a leading cause of global morbidity and mortality, ranking second only to cardiovascular diseases (Siegel et al. 2024). Conventional cancer treatments, including surgery, chemotherapy, radiotherapy, immunotherapy, hormone therapy, and targeted therapies, remain essential for disease management. Among these, chemotherapy and radiotherapy are the most widely applied due to their cytotoxic efficacy against malignant cells. However, their use is frequently limited by severe side effects such as fatigue, alopecia, gastrointestinal complications, and skin damage, as well as by tumor recurrence and the development of multidrug resistance (Gavas et al. 2021; Wiraswati et al. 2025). Natural products and their derivatives have become attractive candidates, offering potent biological activity with lower systemic toxicity. Indeed, approximately 60% of clinically approved anticancer agents are derived from natural sources (Wiraswati et al. 2025; Newman and Cragg 2020). Marine natural products, though less explored than their terrestrial counterparts, represent an invaluable reservoir of bioactive compounds (Palanisamy et al. 2017). Among these, bromophenols (BPs**)** from marine algae, such as *Rhodomela confervoides*, *Leathesia nana*, and *Polysiphonia lanosa*, have attracted attention for antioxidant and anticancer properties (Goya and Mateos 2024; Barciela et al. 2024; Carpena et al. 2024; Xu et al. 2004a; Duan et al. 2007; Dong et al. 2020). Several studies have demonstrated the anticancer potential of natural and synthetic bromophenols (Shi et al. 2009; Popplewell and Northcote 2009; Colon et al. 1987). However, some bromophenolic compounds display limited selectivity toward non-cancerous cells (Han et al. 2005; Xu et al. 2004b). To enhance selectivity, solubility, and pharmacological efficacy, strategies such as structural modification and advanced drug delivery systems are increasingly being investigated. Nanotechnology-based drug delivery systems have emerged as powerful tools in oncology, addressing solubility, stability, and non-specific toxicity (Dadwal et al. 2018). Among them, liposomes—spherical vesicles consisting of phospholipid bilayers—have become particularly prominent due to biocompatibility, versatility, and encapsulation ability (Rommasi and Esfandiari 2021; Sapra et al. 2005). Liposomes were discovered by Bangham and colleagues in the 1960s, and their optimized sizes (50–450 nm) enable effective tumor accumulation through the enhanced permeability and retention (EPR) effect (Etheridge et al. 2013). Surface modifications such as PEGylation and cholesterol incorporation improve circulation time and stability, while ligand-functionalized liposomes enable targeted delivery (Nakhaei et al. 2021; Fulton and Najahi-Missaoui 2023; Wang et al. 2023; Hasan et al. 2020). Liposomal formulations have demonstrated clinical potential in cancer therapy (Fanciullino and Ciccolini 2009). In this study, we aimed to encapsulate a newly synthesized bromophenolic compound (BP), structurally similar to bioactive marine bromophenols, and the model drug etoposide into PEGylated liposomes prepared using a thin-film hydration technique. The antitumor and proapoptotic activities of free BP and liposomal bromophenol (LipoBP) were evaluated in A549 and MCF-7 cancer cell lines alongside the model drug etoposide through cytotoxicity, apoptosis, and gene expression analyses. To explicitly highlight the novelty of this work, the bromophenol used in our study is a newly synthesized diaryl bromophenol derivative, inspired by marine natural bromophenols, and has not been reported in any previous anticancer research. Its unique structural features, including a brominated diphenylmethane scaffold, were designed to enhance bioactivity and selectivity compared to previously studied bromophenols. Moreover, encapsulating a bromophenol in liposomes is a first-of-its-kind approach, as no prior studies have formulated marine bromophenols in liposomal form for cancer therapy. While liposomal delivery has been applied to other plant-derived phenolics (e.g., quercetin, EGCG, fisetin) to improve solubility, stability, and bioavailability (Seguin et al. 2013; Pralhad and Rajendrakumar 2004; Pires et al. 2019; Ragelle et al. 2012), these compounds are structurally distinct from bromophenols. Bromination significantly alters hydrophobicity, membrane affinity, and metabolic stability, making bromophenols fundamentally different (Haldar and Bagchi 2016; Kuramochi et al. 2004; Van Dael and Ceuterickx 1984). Furthermore, natural bromophenols are difficult to obtain in sufficient quantities from marine sources (Dong et al. 2020), so generating a synthetic analogue provides a scalable and practical alternative. The dual novelty of this study—the first synthesis of this bromophenol and its liposomal formulation (LipoBP)—thus fills a clear gap in current phenolic nanodelivery research.

## Materials and methods

### Materials

The main lipid components used for liposome preparation were 1,2-distearoyl-sn-glycero-3-phosphocholine (DSPC; Biosynth, UK), 1,2-distearoyl-sn-glycero-3-phosphoethanolamine-N-[amino(polyethylene glycol)−2000] (DSPE-PEG2000; AmBeed, IL, USA), and cholesterol (Sigma-Aldrich, USA). Etoposide, used as a reference anticancer drug and chloroform were obtained from Merck (Darmstadt, Germany).

The bromophenol compound was synthesized in-house following the procedure reported elsewhere. Its structure was confirmed by 1H and 13 C NMR, IR, and elemental analysis, supporting its identity and structural integrity (Oztaskin et al. 2022). HR-MS and HPLC-based purity analyses were not included within the scope of the present study.

### Methods

#### Preparation of liposomes

**Fig. 1:**
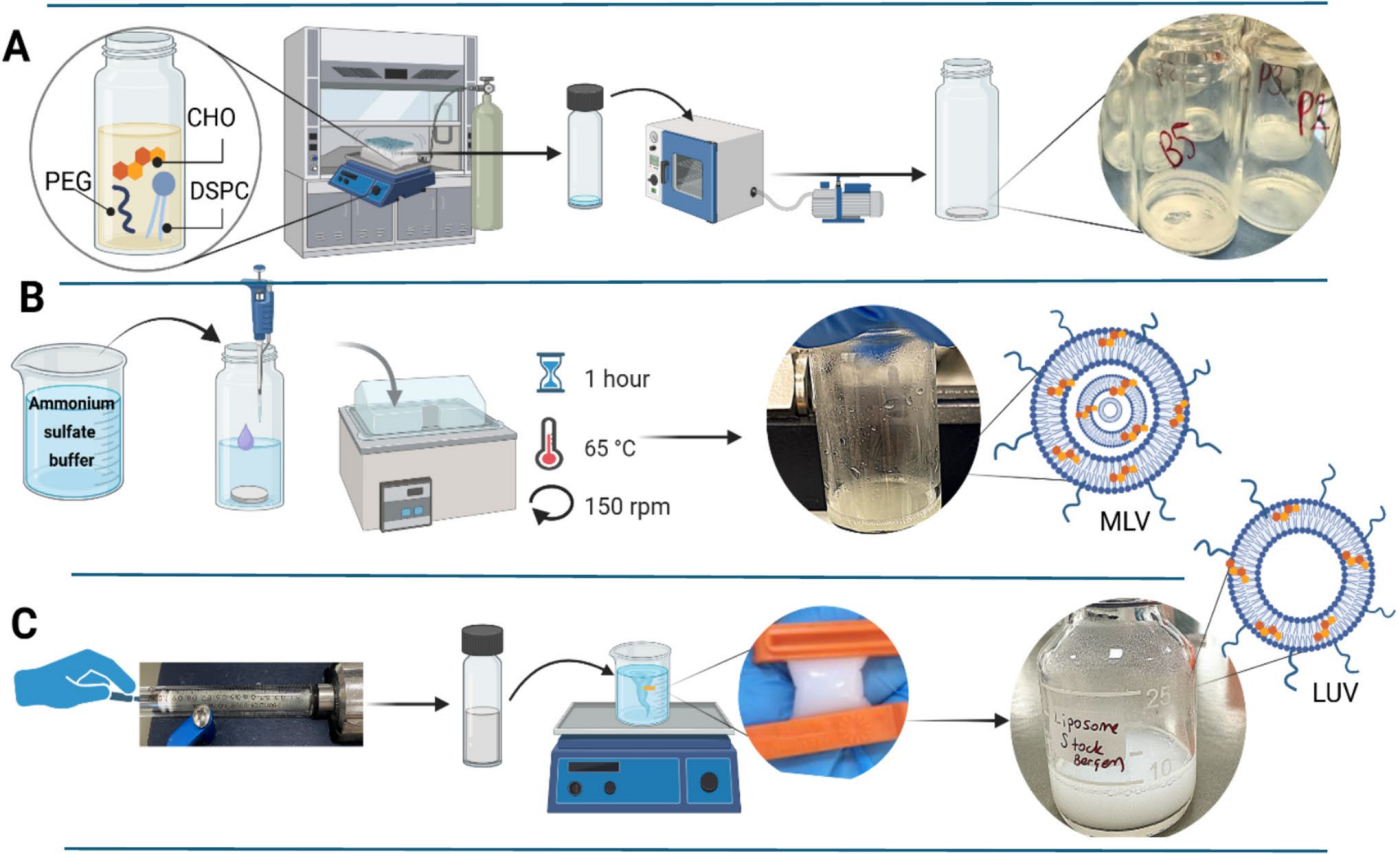
Schematic illustration of liposome preparation by the thin-film hydration method. (**A**) Formation of a uniform lipid thin film; (**B**) hydration of the lipid layer with ammonium sulfate buffer to generate multilamellar vesicles (MLVs); (**C**) downsizing of vesicles by extrusion and removal of excess ions through dialysis. Abbreviations: CHO, cholesterol; DSPC, 1,2-distearoyl-sn-glycero-3-phosphocholine; PEG, DSPE-PEG2000 (amine-1,2-distearoyl-sn-glycero-3-phosphoethanolamine-N-[amino(polyethylene glycol)−2000]); MLV, multilamellar vesicle; LUV, large unilamellar vesicle. *(Figure created by the authors using* https://BioRender.com)

Liposomes were prepared using the conventional thin-film hydration method, as described in our previous study (Hanoğlu 2019). Briefly, as shown in Fig. 1(A), DSPC (9 mg), cholesterol (2.93 mg), and DSPE-PEG_2000_ (2.8 mg) were dissolved in 1 mL chloroform at a molar ratio of 57:38:05. The solvent was evaporated under nitrogen with gentle orbital rotation, and the thin lipid film was further dried under vacuum overnight (Nüve EV 018, Ankara, Turkey) to ensure complete removal of organic residues. As shown in Fig. 1(B), the dry lipid film was hydrated with 1 mL of 250 mM ammonium sulfate ((NH₄)₂SO₄) buffer, pH 5.5, at 65 °C and at 150 rpm with an orbital stirrer for 1 h, to yield multilamellar vesicles (MLVs). DSPC’s gel–liquid crystal transition temperature is about 55 °C (Chen et al. 2018), thus all hydration and extrusion steps were conducted at 65 °C, at above T_m_ to ensure the lipids were in the fluid phase. To obtain large unilamellar vesicles (LUVs), As shown in Fig. 1(C), the MLVs were extruded 11 times through a 200 nm polycarbonate membrane (Whatman®, USA) using a Mini-Extruder (Avanti Polar Lipids, USA) pre-heated to 73 °C, as previously described (Hanoğlu 2019). The liposomal suspension was dialyzed (Spectra/Por® 6, MWCO = 8 kDa) against 0.9% NaCl for 2 h followed by overnight dialysis to remove excess ammonium sulfate. Sink conditions were carefully maintained during dialysis by using a sufficiently large volume of buffer and gentle stirring to avoid accumulation of released solutes.The resulting liposomes were stored at 4 °C until use.

#### UV characterization of bromophenol

The absorption spectrum of bromophenol (BP) was recorded using a UV–Vis spectrophotometer (200–600 nm) as previously reported for phenolic compounds (Mutiarahma et al. 2021). Calibration curves were established to determine the extinction coefficient and evaluate BP solubility in water, chloroform, Acetonitrile, and DMSO. The wavelength of maximum absorbance (λ_max_) was used in subsequent quantitative assays.

#### Drug loading and determination of encapsulation and loading efficiency

**Fig. 2:**
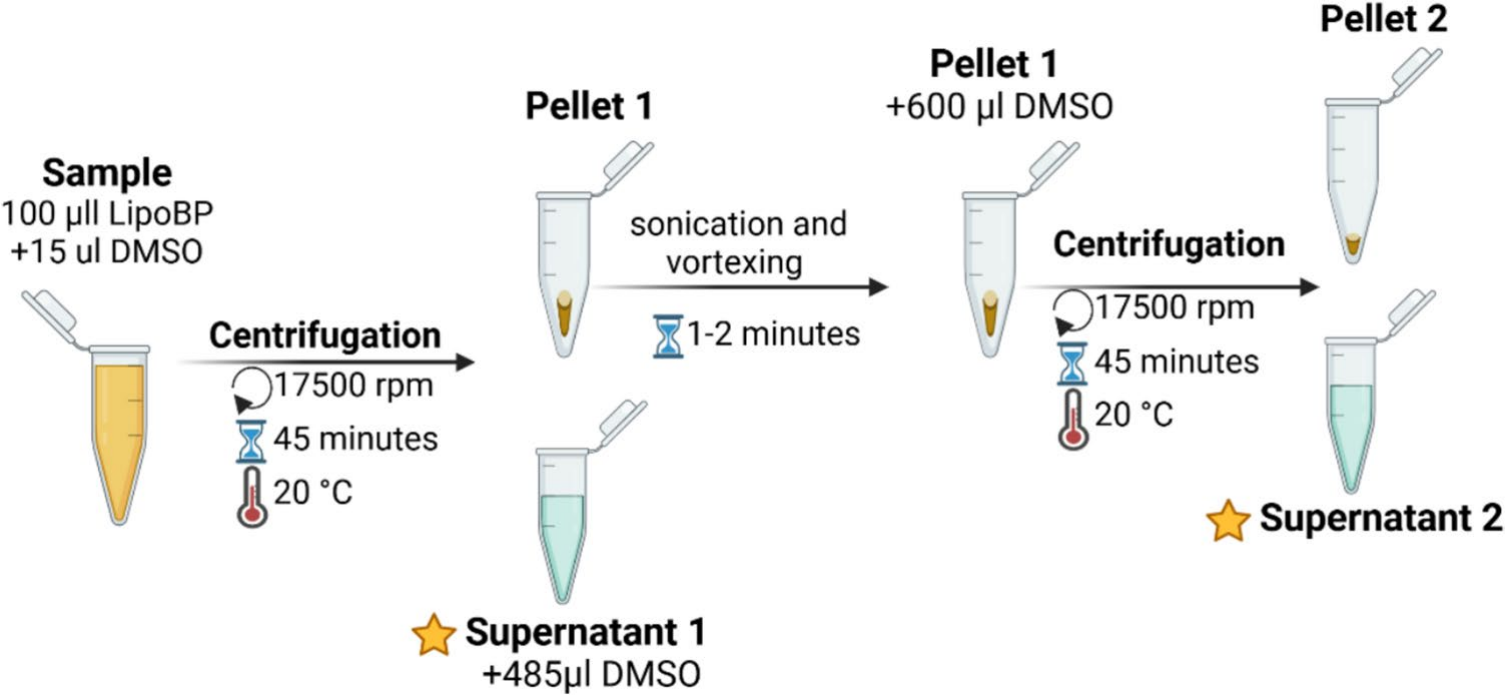
Schematic representation of the procedure for determining bromophenol (BP) content using the centrifugation method

BP was incorporated into the liposomal bilayer using two separate strategies: passive loading and active (remote) loading. Passive loading was first attempted to allow spontaneous insertion of the hydrophobic drug into the lipid bilayer; however, this approach resulted in poor retention, consistent with reports for small hydrophobic compounds. Subsequently, active loading based on a transmembrane ammonium sulfate gradient was employed, which proved to be more efficient and reproducible, in line with previous literature (Haran et al. 1993; Lee et al. 2025, 2005; Presa et al. 2009; Seguin et al. 2013; Barenholz and Peer 2012; Danaei et al. 2018). Stock solutions of BP and etoposide (control) were prepared in DMSO at 5 mg/mL. For active loading, after forming empty liposomes, the DMSO-based drug solution was added at 16% of the total mixture volume, an optimized ratio that maximized drug loading while maintaining liposome stability. The mixture was incubated in a shaking water bath (65 °C, 150 rpm) for 2 h. Unencapsulated drug was removed by dialysis as described previously. Encapsulation efficiency (EE%) and loading efficiency (LE%) were determined using the high-speed centrifugation method, as shown in Fig. 2 (Kim et al. 2022). After centrifugation (17,500 rpm, 20 °C, 45 min), liposomes were pelleted, and the supernatant (unencapsulated drug) was collected. A 115 µL aliquot of the supernatant was diluted with 485 µL DMSO (supernatant 1). The liposomal pellet was redissolved in 600 µL DMSO, sonicated, and re-centrifuged to obtain supernatant 2. UV–Vis absorbance (200–600 nm) of both fractions was measured to calculate EE and LE using Eqs. (1) and (2).

Encapsulation efficiency (EE) and Loading efficiency (LE) of LipoBPs was calculated based on the following two equations (Önol 2023).

Loading efficiency (LE%) was calculated as

1 $$\mathrm{LE}\left(\%\right)=\frac{{\mathrm{W}}_{\mathrm{i}}}{{\mathrm{W}}_{\mathrm{S}}}$$

where, W_i_ is drug entrapped within liposome, and W_S_ is inially added drug to the liposomal formulation.

Encapsulation efficiency (EE%) was calculated as

2 $$\mathrm{EE}\left(\%\right)=\frac{{\mathrm{W}}_{\mathrm{i}}}{{\mathrm{W}}_{\mathrm{T}}}$$

where, W_i_ is loaded drug or internal drug amount within the liposomes, and W_T_ is the total of internal (W_i_) and external (W_e_) drug amount estimated for the liposomes. Liposomal etoposide was prepared in parallel using the same procedure, to serve as a directly comparable formulation.

#### Characterization of liposomes

Particle size, polydispersity index (PDI), and zeta potential were determined by dynamic light scattering (DLS) using a Zetasizer Nano-ZS (Malvern Instruments, UK). Liposome dispersions were diluted (20 µL sample + 980 µL 10 mM NaCl) and equilibrated for 60 s before measurement. All data represent mean ± SD of triplicate measurements at 25 °C. Independent batches were prepared for each experimental section: one batch for liposome characterization, one batch for liposomal formulation and drug encapsulation efficiency, one batch for release studies, and a single batch was used collectively for cytotoxicity, apoptosis, and gene-expression in vitro studies.The stability of plain and BP-loaded liposomes was evaluated at 4 °C for up to 10 days (plain) and 3 days (loaded). At specific intervals, samples were diluted (20:980 µL, v/v) with 10 mM NaCl and analyzed by DLS for hydrodynamic diameter, PDI, and zeta potential. To assess BP chemical stability, UV–Vis absorbance of aqueous BP solutions was measured at Day 0 and Day 3. In line with typical nanocarrier development standards, this short-term stability assessment was designed to monitor early physicochemical integrity following formulation; however, long-term storage stability was beyond the scope of this study and will require future investigation.

#### Drug release from liposomes

The drug release profiles of LipoBP and liposomal etoposide were tested at 37 °C and 43 °C, respectively. Drug releases were assessed at 37 °C, physiological, and at 43 °C, to examine the effect of mild hyperthermia. Temperatures in the 40–43 °C range are commonly used to trigger drug release from thermosensitive liposomes (Ta and Porter 2013; Chen et al. 2018), so testing at 43 °C can reveal any potential temperature-responsive release behavior of our formulation. The procedure in Fig. 2 along with Eq. 1 and Eq. 2 were used for evaluations. Samples were immersed in a gentle shaking environment at the temperatures mentioned, and 100 µL aliquots were withdrawn at specific time points. Release profiles were expressed by spectrophotometrically measuring the time course of internal and external drug amounts for each sample. A Spectra/Por® 6 dialysis membrane (MWCO = 8 kDa) was used; both etoposide (~ 588.56 Da) (Merck Index. (n.d.). Etoposide. In The Merck Index Online. Royal Society of Chemistry 2025) and the bromophenol derivative (~ 374 Da, estimated from chemical structure) are well below this cutoff, minimizing potential membrane-imposed diffusion limitations. Sink conditions were maintained, and gentle shaking was applied throughout the experiment to ensure consistent diffusion and accurate measurement of true drug release.

#### Cell culture

Human breast adenocarcinoma (MCF-7) and lung carcinoma (A549) cell lines were obtained from the American Type Culture Collection (ATCC, USA). MCF-7 cells were cultured in Dulbecco’s Modified Eagle Medium (DMEM, high glucose), and A549 cells in RPMI-1640 medium, each supplemented with 10% (v/v) fetal bovine serum (FBS; EuroClone, Italy) and 1% penicillin–streptomycin. Cells were maintained at 37 °C in a humidified atmosphere containing 5% CO_₂_ and were subcultured two to three times per week.

#### In vitro cytotoxicity assay

Cytotoxicity of free BP, LipoBP, free etoposide (ETP) and liposomal etoposide (LETP) was assessed using the WST-8 assay (Cell Counting Kit-8; Abbkine, USA) (Lee et al. 2005). MCF-7 and A549 cells (1 × 10^5^ cells/well) were seeded in 96-well plates and incubated for 24 h before treatment. Test samples (200–3,375 µg/mL) were added and incubated for 24 h, followed by 10 µL CCK-8 reagent per well. After 1–4 h, absorbance was read at 450 nm (Thermo Scientific Multiskan GO, USA). Cell viability and IC₅₀ values were calculated from nonlinear dose–response curves. BP and etoposide stock solutions were prepared in DMSO and diluted into the culture medium, resulting in a final DMSO concentration of 0.5% for all treatments, which is a low concentration commonly used in in vitro studies and generally expected to have minimal cytotoxic effects (Galvao et al. 2014). Therefore, the observed cytotoxicity in this study is attributable to the active compounds rather than the carrier or solvent. All liposomal formulations were prepared in isotonic 0.9% NaCl, and because stock solutions were added in minimal volumes, significant osmolarity changes were not expected.

#### Apoptosis assay

Apoptotic induction was quantified by Annexin V-FITC/PI staining (ABP Biosciences, USA) and flow cytometry. MCF-7 and A549 cells (3 × 105 cells/well) were seeded in 6-well plates and treated with plain liposomes (PL), free bromophenol (BP), LipoBP (LBP), free etoposide (ETP) or liposomal etoposide (LETP) at their IC₅₀ values for 48 h. A negative control group, containing only culture medium, was included. Harvested cells were washed with cold PBS, stained with 5 µL Annexin V-FITC and 2 µL PI in 100 µL binding buffer, and incubated for 15 min in the dark. After adding 400 µL binding buffer, fluorescence was analyzed using a Beckman Coulter CytoFLEX flow cytometer (excitation 488 nm; emissions 530 nm and > 575 nm).

To accurately determine apoptosis, a sequential gating strategy was applied. First, cellular debris was removed using FSC/SSC parameters. Then, using FSC-A and FSC-H graphs, double cells and cell aggregates were eliminated to select the single-cell population. Apoptosis analysis was performed on this single-cell population using Annexin V-FITC and PI fluorescence signals. Quadrant gates were defined as follows according to the standard interpretation of Annexin V/PI staining:

- Annexin V⁻/PI⁻: live cells
- Annexin V⁺/PI⁻: early apoptotic cells
- Annexin V⁺/PI⁺: late apoptotic cells
- Annexin V⁻/PI⁺: necrotic cells

To correct for spectral overlap between the FITC and PI channels, fluorescence compensation was adjusted using single-stained control samples, and instrument parameters were optimized prior to sample analysis. Data collection and analysis were performed using CytExpert software (Beckman Coulter). The same gating strategy and quadrant settings were consistently applied across all experimental groups and both cell lines. Representative dot plot images for MCF-7 and A549 cells are shown in Fig. 10. Apoptosis percentages were expressed as the sum of early and late apoptotic cell populations. Data are presented as the mean ± SD (n = 3) from three independent experiments, each with three replicates.

#### Gene expression analysis

Expression of *BAX*, *BCL-XL*, *CASP6*, *IQSEC2, CASP3* and *CASP9* was quantified by qRT-PCR following established protocols. Cells were treated with plain liposomes (PL), free bromophenol (BP), or liposomal Bromophenol (LBP). Total RNA was extracted using the EcoPURE Total RNA Kit (ECOTECH Biotechnology, Turkey), and cDNA was synthesized using the High Capacity ABT™ cDNA Synthesis Kit (Ankara, Turkey). qRT-PCR was performed using a Rotor-Gene Q (Qiagen, Germany) and ClearPeak 2X SYBR Probe Master Mix (ECOTECH). The thermal profile consisted of 95 °C for 30 s; 40 cycles of 95 °C for 10 s, 60 °C for 30 s, and 72 °C for 20 s. All qPCR assays were confirmed to produce a single product, melt-curve analysis showed one peak for each primer pair, verifying specificity of amplification. Primers are listed in Table 1. Relative gene expression was analyzed via the 2⁻ΔΔCt method with β-actin as the reference gene (Livak and Schmittgen 2001).

**Table 1:** Genes and primers used in qRT-PCR

Gene	Forward Primer (5′–3′)	Reverse Primer (5′–3′)	NCBI ID
*BAX*	ACCGTGACCATCTTTGTGG	CTCAGCCCATCTTCTTCCAG	NM_001291430.2
*CASP6*	GCCCCGTCTCTACTAAAAATG	AGTGATTCTCCTGCCTCAGC	NR_133012.2
*BCL-XL*	CATCCCTACCCCCTAAGAGC	TATCCCAAGCAGCCTGAATC	NM_001317921.2
*IQSEC2*	TTCCCCTGCTTACCATTGAG	AGCCATCAGCCTCATACACC	NM_001441094.1
*CASP3*	AGTAGATGGTTTGAGCCTGAGC	AGTGCGTATGGAGAAATGGG	NM_004346.4
*CASP9*	ATATCTAGTTTGCCCACACC	CCCTTTCACCGAAACAGCAT	NM_001229.5
*Β-ACTIN*	TCTTTTCCAGCCTTCCTTCC	CATACAGGTCTTTGCGGATG	HQ154074.1

*F: Forward, R: Reverse

#### Statistical analysis

Data are presented as mean ± standard deviation (SD) of three independent experiments. Statistical significance was determined using one-way ANOVA followed by Tukey’s post hoc test. IC₅₀ values were obtained from nonlinear regression analysis using GraphPad Prism v10.4.2 (GraphPad Software, USA). Significance levels: ns (p > 0.05), * (p < 0.05), ** (p < 0.01), *** (p < 0.001), **** (p < 0.0001).

## Results and discussion

### UV–Vis characterization of BPs

**Fig. 3:**
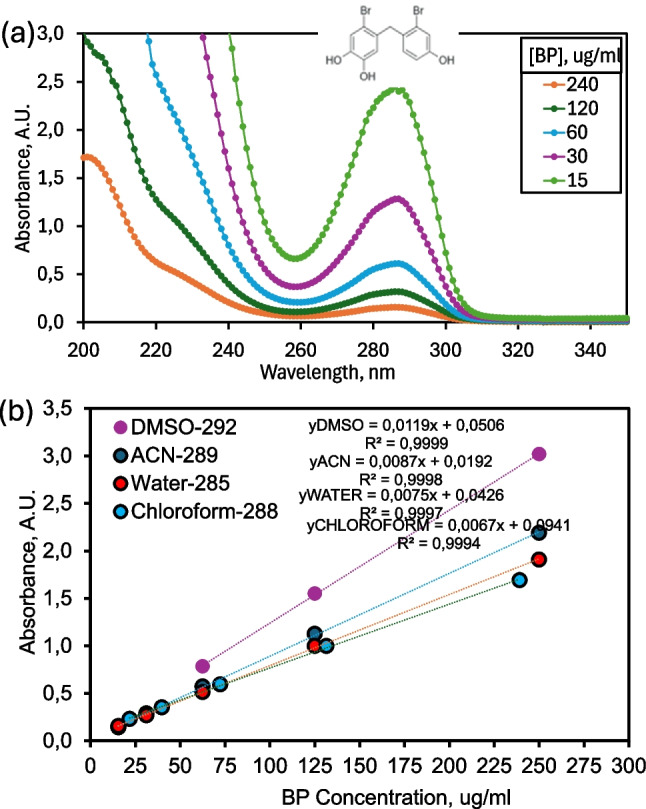
(**a**) UV–Vis absorbance spectrum of bromophenol (BP) in aqueous solution, (**b**) calibration curve illustrating the linear relationship between absorbance and BP concentration at 285 nm, and comparison of the maximum absorbance wavelengths of BP in aqueous and DMSO solutions, demonstrating the solvent-dependent spectral shift

UV–Vis spectroscopy was used for the detection, quantification and structural confirmation of bromophenol (BP) and etoposide, with etoposide serving as a model anticancer drug. This technique has also been reported to be preferred in previous studies for purposes such as determining the proven antioxidant activities of natural bromophenols (Han et al. 2005) and synthetic bromophenol derivatives (Öztaşkın et al. 2015, 2019). The absorbance–concentration plots of BP were shown in Fig. 3(b) in aqueous and other organic solutions. Spectroscopic analysis of BP revealed a maximum absorbance wavelength (λmax) at approximately 285 nm in aqueous solution (Fig. 3a), while bromophenol was observed not to exhibit fluorescence (data not shown). Calibration curves showed linearity (R^2^ > 0.99), indicating a direct relationship between absorbance and BP concentration (Fig. 3b). The λmax value of BP varied between 285–292 nm depending on solvent polarity and was slightly shifted towards longer wavelengths (bathochromic shift) in DMSO compared to water. Such solvent-dependent spectral shifts are common among brominated phenolics and indicate enhanced π–π* electronic transitions in less polar environments (Haldar and Bagchi 2016). As expected, the solubility of BP in water was significantly lower than in DMSO due to hydrophobic bromine substituents, which reduce molecular polarity and hydrogen bonding capacity (Kuramochi et al. 2004). While phenolic hydroxyl groups provide partial aqueous solubility, the presence of bromine atoms makes the molecule amphiphilic, which explains its preferential incorporation into lipid bilayers (Van Dael and Ceuterickx 1984). In liposomal systems, limited aqueous solubility can be advantageous because hydrophobic molecules preferentially associate with the lipid phase, improving encapsulation and retention (Akaki et al. 2024). DMSO, a solvent commonly used to dissolve hydrophobic drugs (Da Violante et al. 2002; Baybekov et al. 2021), increased the solubility and spectral clarity of BP. These findings are consistent with previous studies reporting similar UV absorption behavior for synthetic bromophenol derivatives and marine phenolics with antioxidant and anticancer properties (Matulja et al. 2022; Balaydın et al. 2010).

### Liposomal formulation and drug encapsulation efficiency

**Fig. 4:**
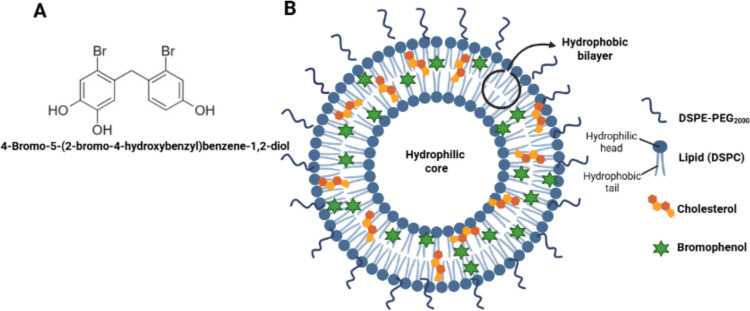
(**A**) Chemical structure of the synthesized bromophenolic compound (BP) and (**B**) schematic illustration of the bromophenol-loaded liposomal formulation. (Created by the authors using BioRender.com.)

Liposomal formulations were designed to encapsulate BP and etoposide within the lipid bilayer, exploiting their hydrophobic character. Chemical structure of the synthesized bromophenolic compound in Fig. 4(A) and the designed liposomal drug delivery platform is depicted in Fig. 4(B). As shown in the Fig. 4(B), the hydrophobic drugs were intended to be loaded within the bilayer of the liposomes. PEG chains were incorporated to prolong liposome circulation in vivo. Liposome size was controlled at ~ 200 nm using polycarbonate membranes with predetermined pore sizes.

**Fig. 5:**
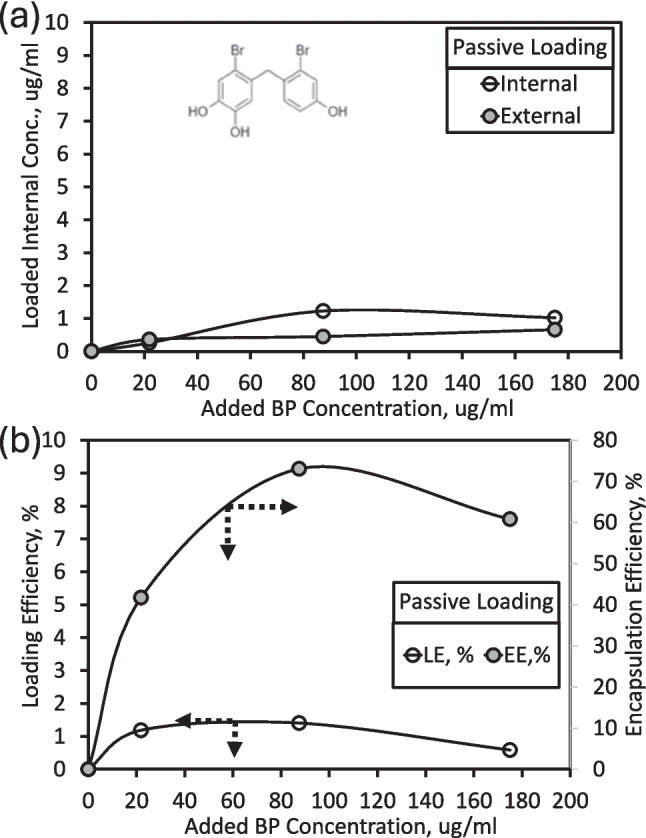
(**a**) Schematic representation of bromophenol (BP) loading within liposomes and (**b**) quantitative evaluation of drug encapsulation efficiency (%EE) and loading efficiency (%LE)

Bromophenol and etoposide were loaded into liposomes using two strategies. Passive loading involved direct mixing of drugs with lipids followed by extrusion, whereas active loading utilized a pH or ion gradient. Passive loading resulted in poor drug retention: internal and external bromophenol concentrations were ~ 1 µg/mL, with loading efficiency (LE) below 3% (Fig. 5). This aligns with literature, which indicates that passive encapsulation often leads to leakage and low entrapment, particularly for small hydrophobic compounds (Lee et al. 2025).

**Fig. 6:**
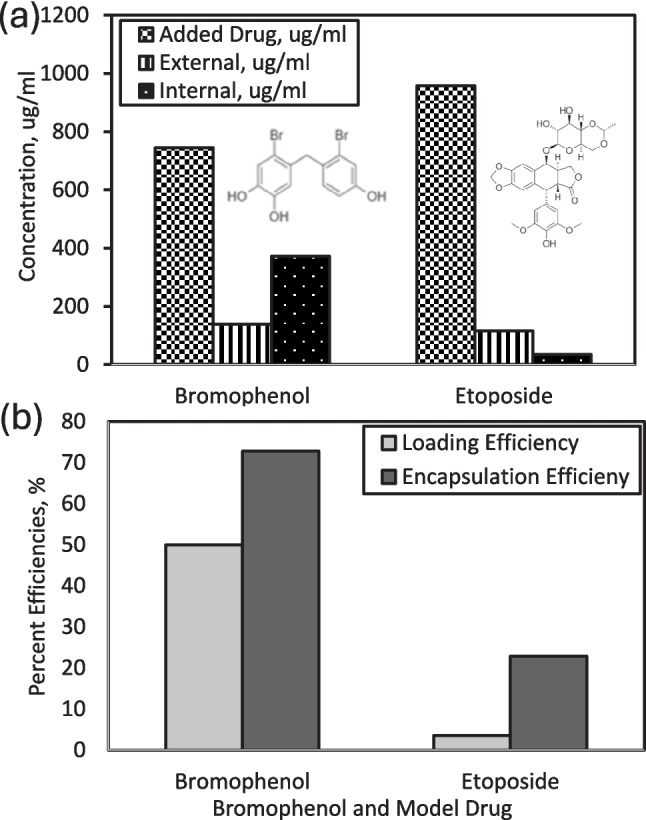
(**a**) Chemical structures and relative concentrations of bromophenol (BP) and etoposide incorporated inside and outside the liposomes, and (**b**) comparison of encapsulation efficiency (%EE) and loading efficiency (%LE) for liposome-loaded formulations

Active (remote) loading significantly improved encapsulation. Molecular structures of bromophenol and etoposide, along with their intra- and extra-liposomal concentrations, are presented in Fig. 6(a). Active loading achieved a loading efficiency of ~ 50% and an encapsulation efficiency (EE) of ~ 73% for BP (Fig. 6b), whereas etoposide reached only ~ 4% LE and ~ 23% EE due to its bulkier, partially hydrophilic structure, which limits incorporation into the lipid bilayer (Lee et al. 2025, 2005; Presa et al. 2009).

The superior encapsulation of BP is attributed to its smaller molecular size and higher hydrophobicity, facilitating insertion between phospholipid tails (Lee et al. 2005; Presa et al. 2009). Similar trends have been reported for other phenolic compounds, such as quercetin and fisetin, which exhibit improved entrapment in PEGylated liposomes compared to more polar drugs (Seguin et al. 2013). These findings emphasize the critical role of molecular structure, hydrophobicity, and steric compatibility in determining encapsulation efficiency, corroborating earlier reports by Barenholz (Barenholz and Peer 2012) and Danaei et al. (Danaei et al. 2018) on structure–lipid interactions during liposome formation.

### Physicochemical characterization of liposomes

Dynamic light scattering (DLS) analysis revealed that both plain and drug-loaded liposomes maintained mean diameters around 200 ± 15 nm, with polydispersity indices (PDI) near 0.3, indicating good homogeneity suitable for pharmaceutical applications (Danaei et al. 2018). These values remained stable over the monitored short-term period (up to 10 days for plain and 3 days for drug-loaded liposomes), confirming that DSPC-based PEGylated liposomes provide initial physical stability (Table not shown). Zeta potential measurements showed nearly neutral surface charges (–8 ± 3 mV). Although zeta potentials outside ± 30 mV usually indicate electrostatic stabilization, PEGylation and sterol incorporation provide steric stabilization, preventing aggregation even at near-neutral potentials (Németh et al. 2022). This observation is in agreement with prior studies on PEGylated DSPC liposomes, which demonstrated high colloidal stability and long circulation half-life (Haeri et al. 2014; Cao et al. 2022). Overall, the stability and uniform particle size of the formulations support their suitability for further biological evaluation, although long-term physical stability—which is critical for translational application—will need to be assessed in future studies. Similar particle size, PDI, and zeta potential values were consistently obtained across independent batches, and mean values are reported to avoid unnecessary repetition.

### Temperature-dependent drug release

**Fig. 7:**
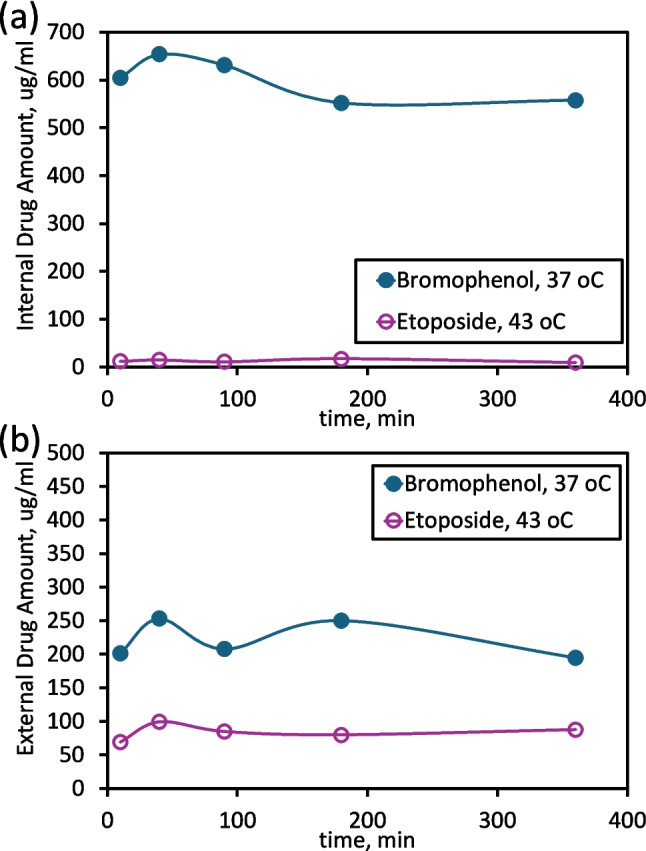
Variation in drug concentration (**a**) retained within and (**b**) released from liposomes over time at different temperatures

Temperature-responsive release was investigated to evaluate the effect of lipid phase behavior on drug diffusion. Figure 7 shows the variation in drug concentration retained within and released from liposomes over time at two temperatures, 37 oC and 43 °C. DSPC lipids possess a high gel–liquid crystalline phase transition temperature (T_m ≈ 55 °C) (Chen et al. 2018), which maintains bilayer rigidity at physiological conditions (37 °C) and limits drug leakage (Kneidl et al. 2014; Ta and Porter 2013; Nagarajan et al. 2012). At 37 °C, bromophenol-loaded liposomes exhibited minimal release (< 10% over 6 h), with internal BP concentrations decreasing slightly from ~ 600 µg/mL to ~ 550 µg/mL, while external concentrations remained around 200–250 µg/mL. These results confirm the strong retention capability of DSPC bilayers in their gel phase (Anderson and Omri 2004; Ullmann et al. 2023). By contrast, etoposide-loaded liposomes exhibited poor encapsulation and higher leakage at 43 °C, corresponding to a phase transition to the liquid-crystalline state. The rapid diffusion observed at elevated temperatures aligns with reports on thermosensitive liposomes, where membrane fluidity enhances drug release (Ta and Porter 2013). Such temperature-responsive behavior can be exploited for localized hyperthermia-triggered drug delivery in oncology (Haemmerich and Motamarry 2018). Thus, BP’s stable encapsulation and controlled release profile suggest that DSPC–PEG liposomes can serve as effective long-circulating carriers for hydrophobic phenolic drugs. The dialysis membrane used (MWCO 8 kDa) is substantially larger than the molecular weights of etoposide (~ 588.56 Da) and the bromophenol derivative (~ 374 Da), ensuring that release kinetics reflect true diffusion. Therefore, the observed release kinetics are unlikely to be affected by membrane limitations, supporting that the release profiles reflect true drug diffusion.

### Cytotoxicity of free and liposomal bromophenol

**Fig. 8:**
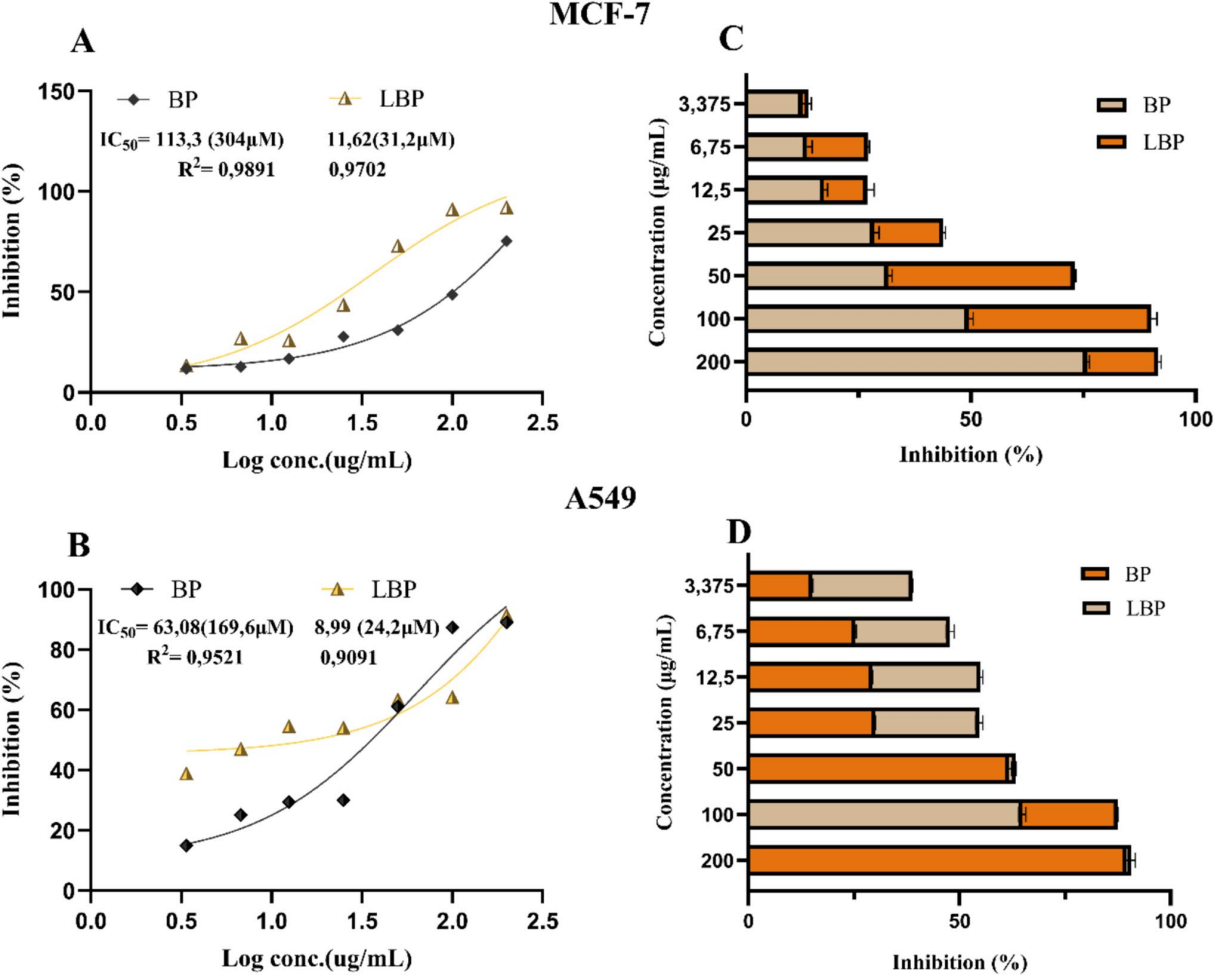
Cytotoxic effects of bromophenol (BP) and liposomal bromophenol (LBP) on cancer cell lines

**Fig. 9:**
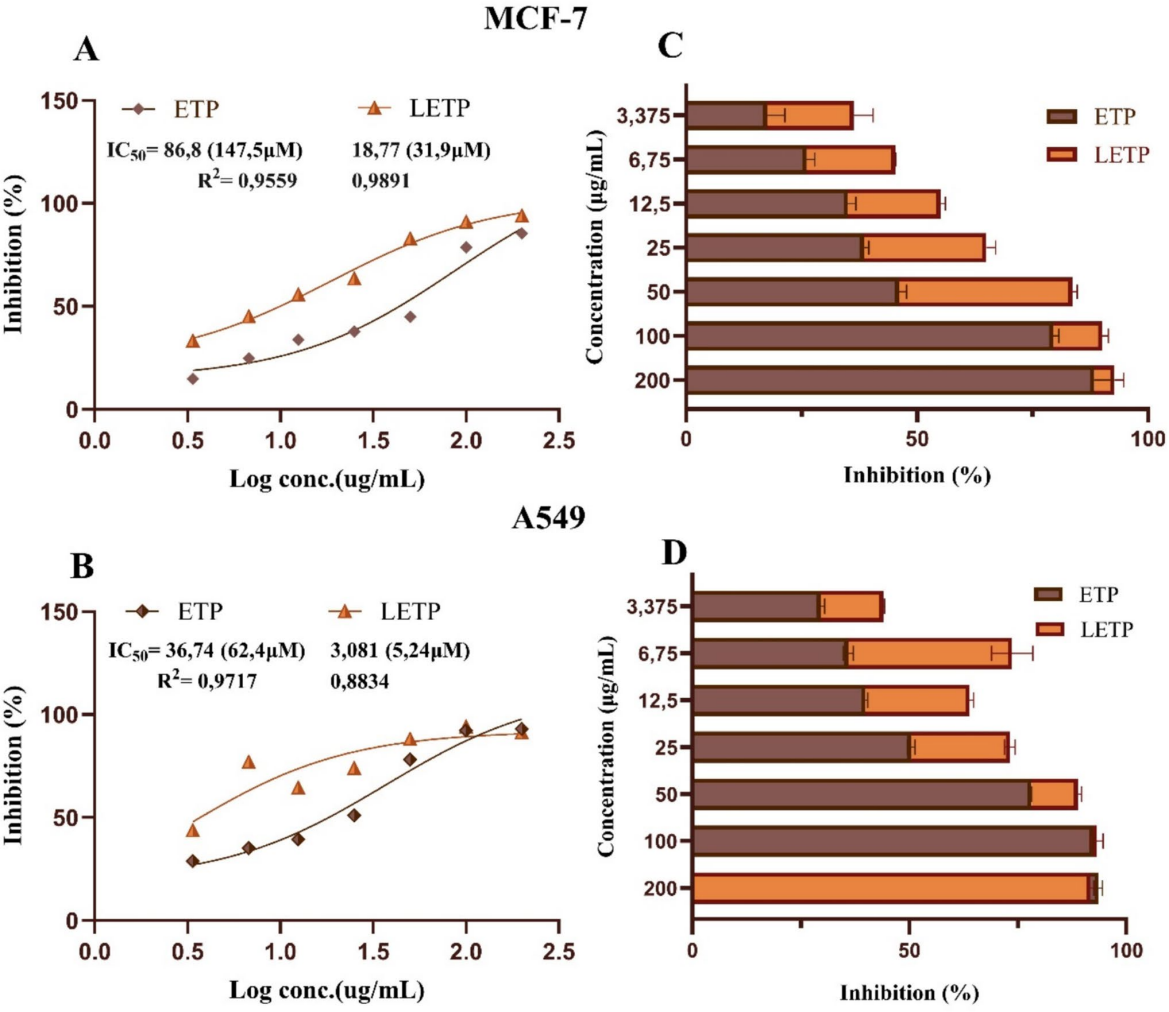
Cytotoxic effects of etoposide (ETP) and liposomal etoposide (LETP) on cancer cell lines

The cytotoxicity of free BP and liposomal BP (LBP) was investigated against MCF-7 (breast) and A549 (lung) cancer cells using the WST-8 assay, as shown in Fig. 8. Both free and liposomal forms of the etoposide were also evaluated and the results were compared (Fig. 9). Free BP and etoposide, were dissolved in DMSO, but the final DMSO content in cell treatments did not exceed 0.2% (v/v) in our WST-8 assays. We included vehicle-only control wells containing the equivalent maximum DMSO concentration. These wells showed no significant effect on cell viability compared to untreated controls. This observation is consistent with literature reports that low DMSO percentages (≤ 0.5%) have minimal cytotoxic impact on most cell lines (Galvao et al. 2014). Both formulations exhibited dose-dependent inhibition of cell viability, with liposomal BP (LBP) showing markedly higher potency—IC₅₀ values of 11.62 µg/mL (MCF-7) and 8.99 µg/mL (A549), compared to 113.3 µg/mL and 63.08 µg/mL for free BP—indicating approximately tenfold (MCF-7) and sevenfold (A549) enhancements. These results suggest that liposomal encapsulation optimizes anticancer effects by improving cellular uptake, stability, and tumor targeting. LETP had the highest activity among the four formulations tested on A549 cells (IC₅₀ 3.081 µg/mL while LBP was the most effective on MCF-7 cells IC₅₀ = 11.62 µg/mL). The obtained cytotoxicity data indicated that the liposomal bromophenol (LBP) formulation we developed exhibited promising cytotoxic activity in comparison with etoposide (ETP), a commercial anticancer agent, in both MCF-7 (breast cancer) and A549 (lung cancer) cell lines. In A549 cells, the IC₅₀ value of LBP (8.99 µg/mL) was approximately 4.1-fold lower than that of free ETP (36.74 µg/mL). Similarly, LBP (IC₅₀: 11.62 µg/mL) displayed approximately 7.5-fold greater activity than free ETP (IC₅₀: 86.8 µg/mL) in MCF-7 cells. These findings suggest that liposomal encapsulation of bromophenol enhances cytotoxic activity and may provide a promising in vitro anticancer effect. The observed activity of LBP can be attributed to improved drug solubility, cellular uptake, and sustained intracellular retention provided by the liposomal carrier (Immordino et al. 2006; Allen and Cullis 2013). PEGylated liposomes are known to facilitate endocytosis-mediated delivery and protect encapsulated drugs from degradation, supporting enhanced anticancer effects (Taher et al. 2023). Comparable enhancements have been reported for other polyphenolic drugs encapsulated in nanocarriers. For example, the water solubility of quercetin, a naturally occurring flavonoid found in plants, was improved by inclusion complexes formed using the freeze-drying technique (Pralhad and Rajendrakumar 2004) and by lipid nanocapsules formed using the phase inversion technique (Barras et al. 2009). Other similar studies include the preparation of simple emulsions of epigallocatechin-3-gallate (EGCG), another flavonoid found in green tea, using the high-pressure homogenization (HPH) technique to design a potential therapeutic agent for cosmetics that would protect against blue light oxidative damage (Pires et al. 2019). The inclusion of fisetin, a natural flavonoid, in nanoemulsions aimed to increase its bioavailability and antitumor activity (Ragelle et al. 2012). Among the limited number of similar studies, the most recent was the study by Seguin et al., which encapsulated fisetin in liposomes to increase its solubility (Seguin et al. 2013). In this study, the anticancer effect of fisetin, a natural flavonoid, was demonstrated, but the practical challenges of its limited water solubility were partially overcome by liposome encapsulation. In fact, in vivo study results demonstrated that the liposomal formulation of fisetin significantly increased the antitumor activity and bioavailability of free fisetin. The similar enhancement observed for bromophenol in this study positions it as a promising lipophilic phenolic candidate for nanocarrier-mediated chemotherapy.

### Apoptosis ınduction

**Fig. 10:**
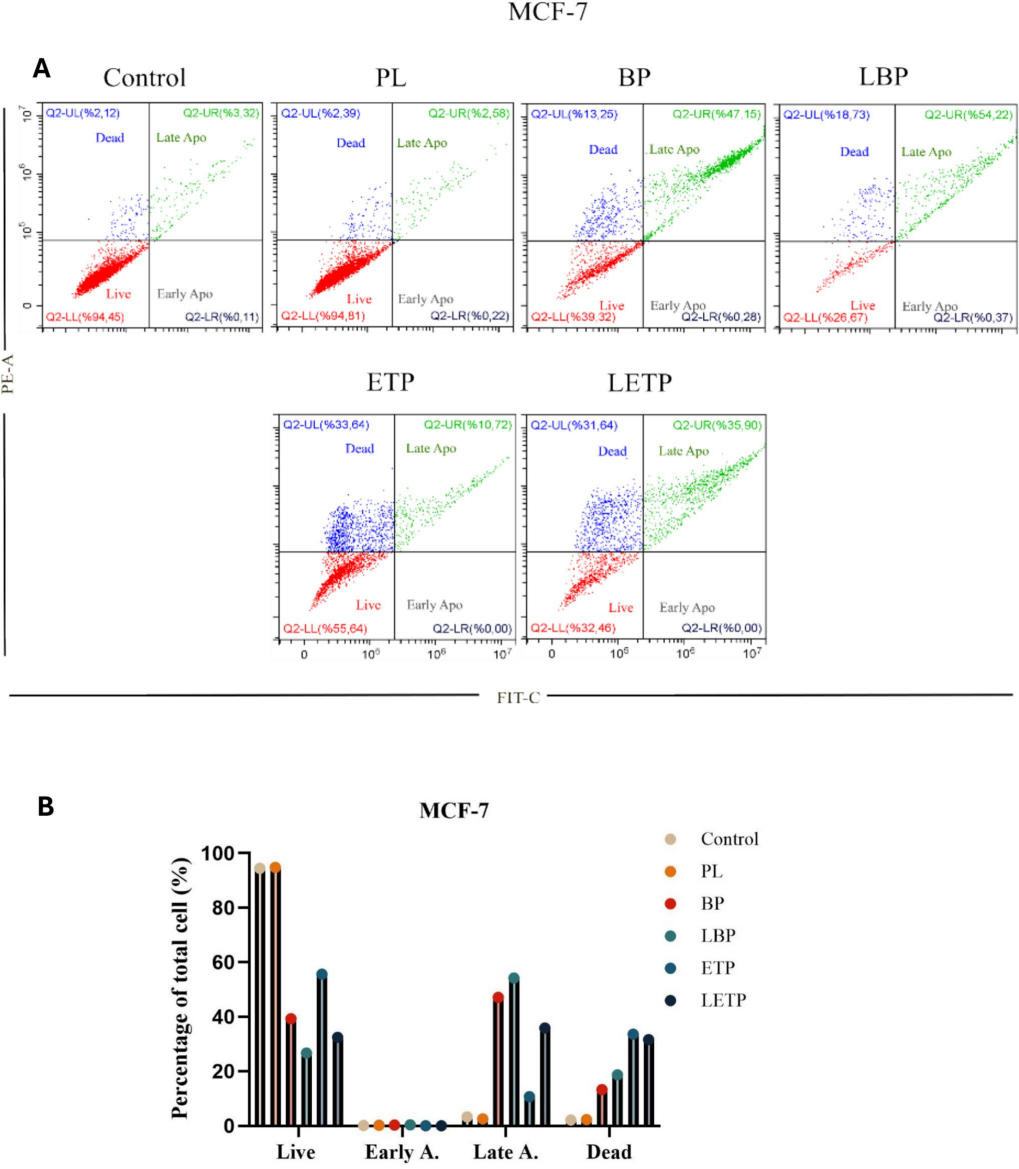

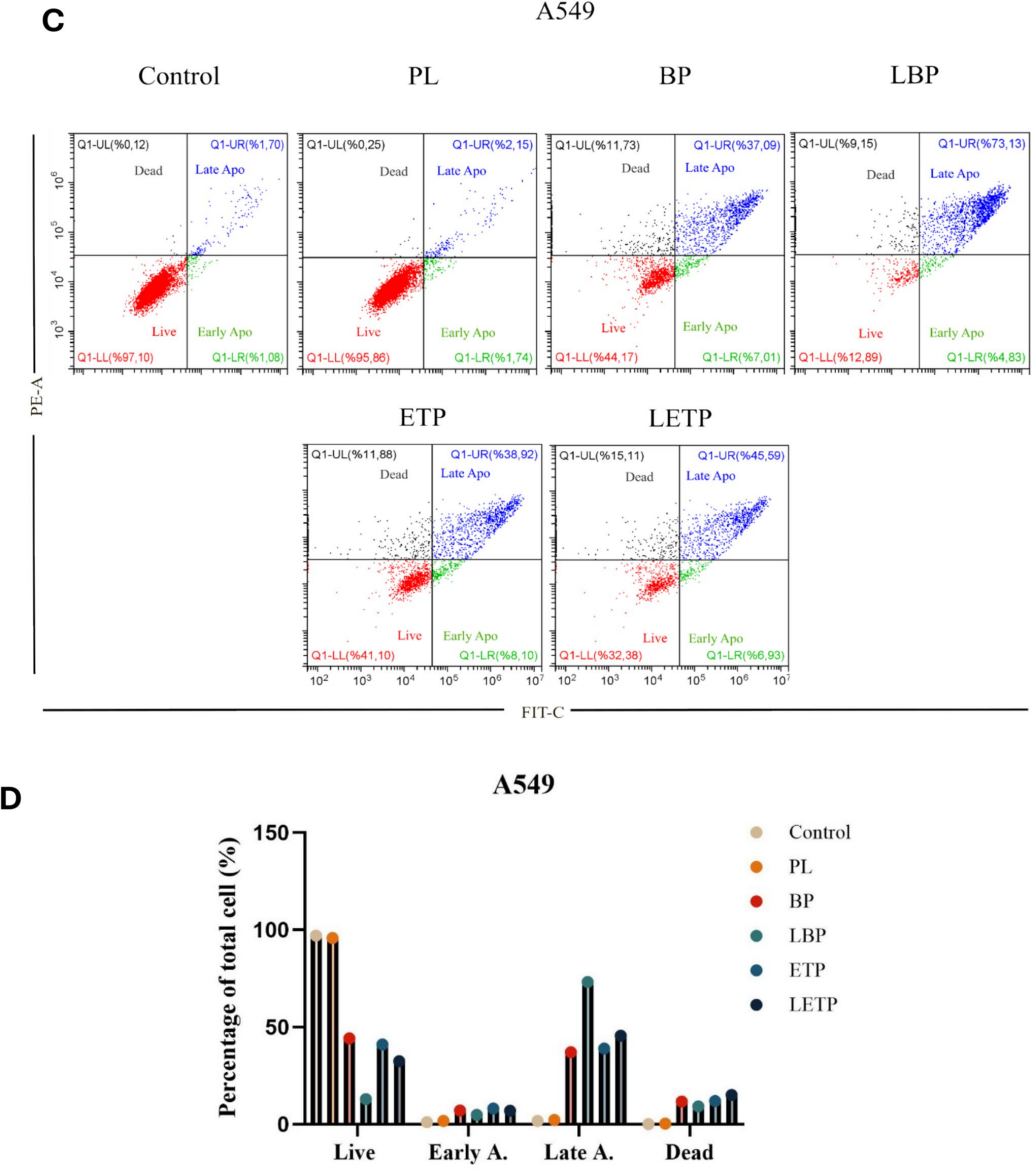
Comparative apoptosis analysis of plain liposome (PL), free bromophenol (BP), free etoposide (ETP), and liposomal etoposide (LETP) formulations in cancer cell lines. (**A**) Apoptosis levels in MCF-7 cells. (**B**) Representative flow cytometry dot plots for MCF-7 cells, showing early apoptosis (Annexin V-FITC⁺/PI⁻), late apoptosis (Annexin V-FITC⁺/PI⁺), and live cells (Annexin V-FITC⁻/PI⁻). (**C**) Apoptosis levels in A549 cells. (**D**) Representative flow cytometry dot plots for A549 cells with the same gating strategy. Untreated cells served as negative controls. Data are presented as mean ± SD from three independent experiments replicated in triplicate (n = 3)

Apoptotic effects were analyzed using Annexin V-FITC/PI staining and flow cytometry as shown in Fig. 10. In our flow cytometry analysis, cells were first gated on forward vs. side scatter to exclude debris and cell aggregates. Then, quadrant gates were set based on unstained and single-stained controls (Annexin V-only and PI-only) to distinguish live, early apoptotic, late apoptotic, and necrotic populations. We did not include these plots in the original manuscript for brevity, but we can confirm that the gating was performed rigorously and consistently for all samples. Annexin V-FITC/PI staining and flow cytometry analysis confirmed the minimal cytotoxicity and high biocompatibility of plain liposomes (PL). Etoposide (ETP), a clinically established anticancer agent, was included as a reference compound; therefore, comparing the apoptotic activity of the newly synthesized bromophenol (BP) and its liposomal form (LBP) with ETP is essential to assess their therapeutic potential within this cellular context.

In MCF-7 cells, the late apoptotic ratios induced by BP (47.15%) and particularly by LBP (54.22) significantly surpassed those observed with free ETP (10.72%) and liposomal ETP (LETP;35.90%). This indicates that BP—especially LBP—elicits a more potent apoptotic response than ETP in this cell line, primarily driving the cells into the final stages of programmed death. In A549 cells, LBP again produced the highest late apoptotic rate (73.13%). Free BP (37.09%) showed slightly lower activity than free ETP (38.92%), suggesting a comparable impact between the non-encapsulated forms in this line. LETP also induced substantial late apoptosis in A549 cells (45.59%), which is significantly higher than its effect in MCF-7 cells (35.90%), highlighting that the A549 line demonstrates a relatively greater sensitivity to ETP compared to MCF-7. These differences may result from cell line–specific factors such as redox balance, uptake mechanisms, and sensitivity to phenolic compounds. Overall, BP—and particularly LBP—induces stronger apoptotic effects than ETP across both cell models, highlighting the formulation advantage of liposomal encapsulation for bromophenol and confirming its preferential promotion of apoptosis in MCF-7 and A549 cells. The enhanced activity of LBP is likely due to improved intracellular delivery and prolonged exposure. This finding is consistent with previous reports showing that liposomal encapsulation of hydrophobic phytochemicals, such as curcumin or resveratrol, enhances mitochondrial apoptosis via reactive oxygen species (ROS) generation and caspase activation (Thayyullathil et al. 2008; Juan et al. 2008). The differential apoptotic responses between A549 and MCF-7 cells may reflect variations in redox state, endocytic capacity, and membrane lipid composition, which influence phenolic compound uptake and apoptosis sensitivity (Pommier et al. 2004).

These trends in enhanced cytotoxicity and apoptotic induction observed for liposomal bromophenol are consistent with previous reports on other nanocarrier systems, where PEG-based micelles improved solubility, sustained release, and cellular uptake leading to increased efficacy (Li et al. 2018; Basu et al. 2024). Additionally, structural optimization of nanocarrier design has been shown to affect release kinetics and therapeutic outcomes in similar nanoparticle platforms (Feng et al. 2024; Yuan et al. 2025), further reinforcing that nanocarrier formulation strategies can broadly enhance the antitumor potential of hydrophobic compounds.

### Gene expression analysis

**Fig. 11:**
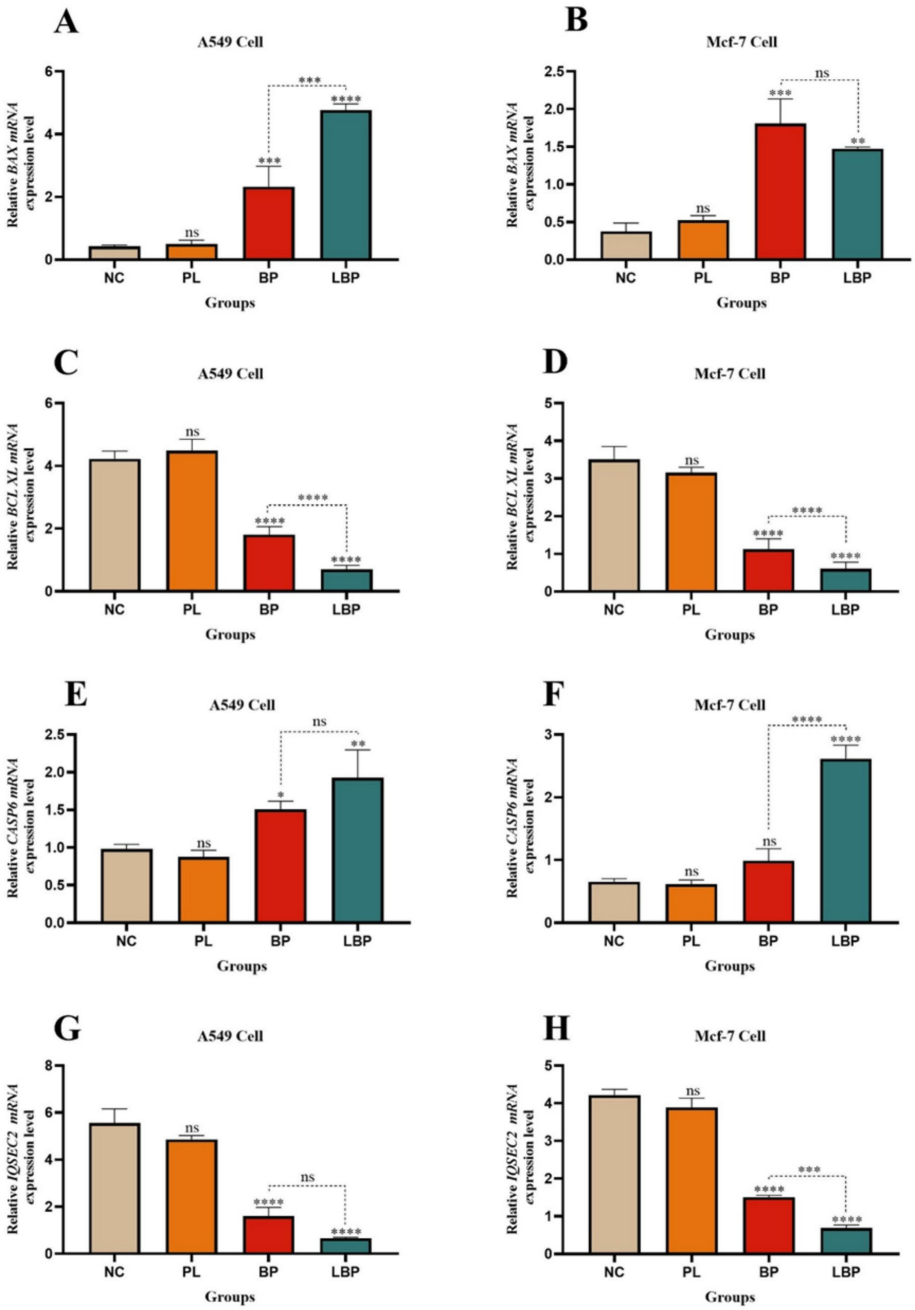

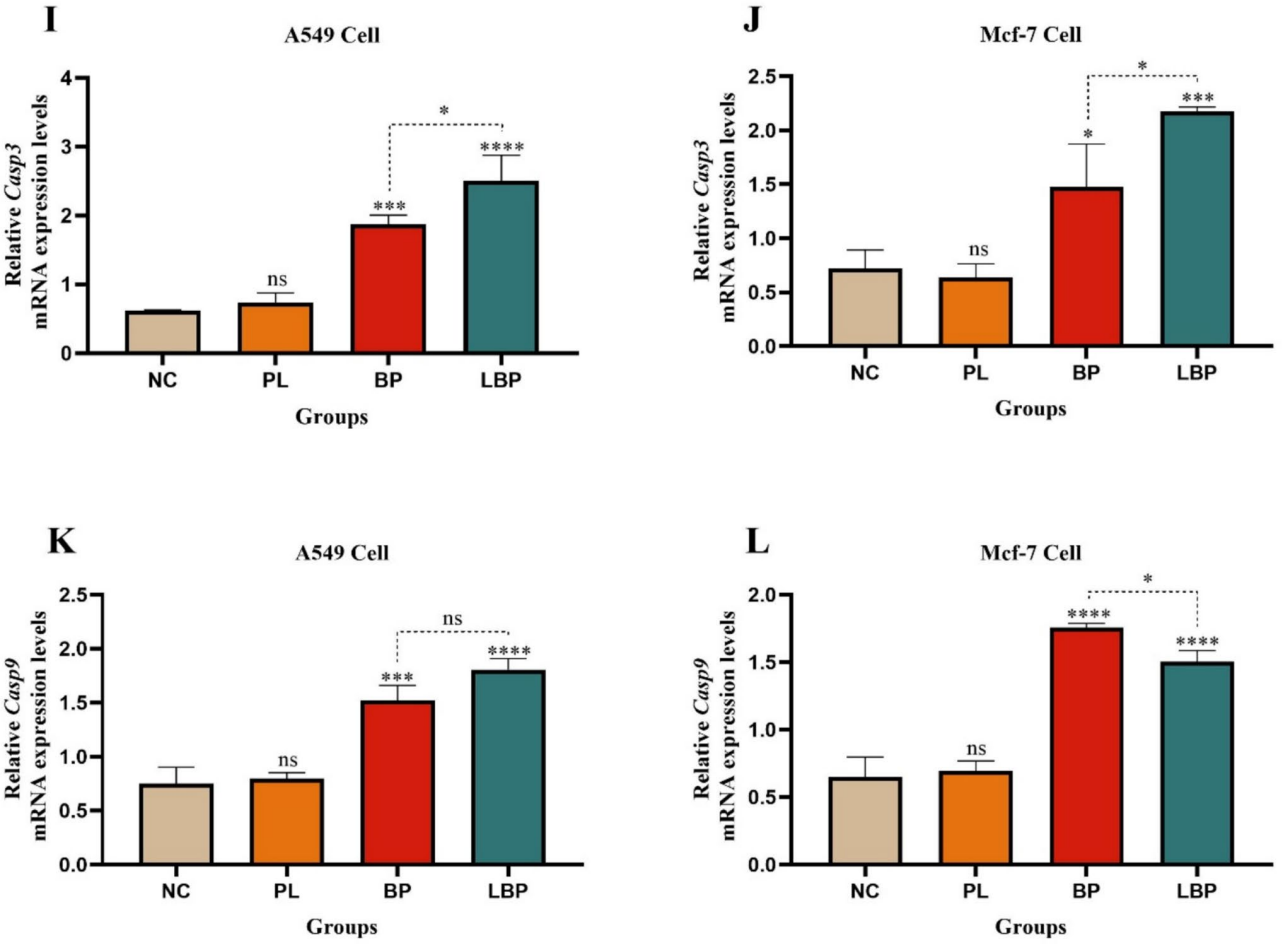
Expression levels of *BAX, BCL-XL*, *CASP6*, *IQSEC2, CASP3* and *CASP9* genes in A549 and MCF-7 cell lines treated with NC (negative control), PL (plain liposome), bromophenol (BP) and LBP (liposomal bromophenol) formulations

To further elucidate apoptotic pathways, expression levels of *BAX*, *BCL-XL*, *CASP6*, *IQSEC2, CASP3* and *CASP9* were examined by qRT-PCR as shown in Fig. 11. In both cell lines, BP and LBP significantly upregulated the proapoptotic *BAX*, *CASP6, CASP3* and *CASP9* genes while downregulating antiapoptotic *BCL-XL* and survival-associated *IQSEC2*. In A549 cells, LBP increased BAX expression approximately fivefold and CASP6 expression 2-fold compared to the control group, while CASP3 and CASP9 expression increased approximately threefold, indicating activation of the intrinsic apoptotic pathway. Meanwhile, *BCL-XL* expression decreased ~ 3.5-fold, and *IQSEC2* decreased ~ 13-fold, highlighting suppression of survival and drug resistance pathways. Similar but less pronounced changes were observed in MCF-7 cells. These gene expression trends align with literature reporting that *BAX*, *CASP6, CASP3* and *CASP9* upregulation promotes mitochondrial membrane permeabilization and caspase activation, leading to apoptotic cell death (Ruiz-Vela et al. 2005; Slee et al. 2001). Conversely, *BCL-XL* overexpression is associated with cancer cell survival and chemoresistance (Janumyan et al. 2003), and *IQSEC2* has been implicated in promoting tumor progression via ARF6-mediated cytoskeletal remodeling (Casalou et al. 2016; Ratcliffe et al. 2019). The pronounced suppression of *IQSEC2* and *BCL-XL* by LBP suggests that liposomal bromophenol disrupts key antiapoptotic and resistance mechanisms, enhancing therapeutic potency. Comparable transcriptional modulation has been observed for other liposome-encapsulated polyphenolics or flavonoids which induce apoptosis through mitochondrial and caspase-dependent pathways (Seguin et al. 2013; Sabaghi et al. 2024). Hence, this study introduces liposomal bromophenol (LipoBP) as a new nanocarrier-based phenolic therapeutic with similar mechanistic characteristics and emphasizes its novelty both in terms of chemical synthesis and liposomal formulation.

Overall, liposomal encapsulation (LipoBP) enhances solubility, stability, cellular uptake, and in vitro antitumor activity of bromophenol, promoting cytotoxicity and apoptosis in both breast and lung cancer cells through upregulation of BAX, CASP6, CASP3, and CASP9, and downregulation of BCL-XL and IQSEC2, thereby activating intrinsic apoptotic pathways and suppressing survival-associated signaling.

To our knowledge, this study is the first to evaluate the liposomal formulation of this newly synthesized bromophenol derivative in cellular models, providing a foundation for future preclinical evaluation and insights into the design of nanocarrier formulations for poorly soluble marine phenolic compounds. However, further mechanistic and long-term characterization will be required to fully establish its translational potential. Although HR-MS and HPLC-based purity analyses were not performed in this study, including them in future work could further strengthen the chemical characterization of this novel bromophenol derivative.

## Conclusion

This study demonstrated that encapsulation of the newly synthesized bromophenolic compound (BP) into PEG-labeled DSPC liposomes (LipoBP) improved its physicochemical stability, bioavailability, and in vitro anticancer performance, suggesting its potential as a promising nanodelivery approach under in vitro conditions. The liposomal strategy effectively addressed the limitations associated with the intrinsic hydrophobicity of BP. Optimized active loading using DMSO enabled the efficient incorporation of BP into the liposomal bilayer, and drug loading and encapsulation efficiencies were found to be increased relative to the reference compound etoposide. The resulting LipoBP formulation exhibited favorable pharmaceutical characteristics—including an average size of ~ 200 nm, low PDI (~ 0.3), near-neutral zeta potential, and temperature-dependent sustained release—supporting its suitability for controlled-release applications. Biological assays showed that LipoBP enhanced the cytotoxic activity of BP in both MCF-7 and A549 cell lines, reducing IC₅₀ values by approximately 7- to tenfold. Comparative analyses further indicated that LipoBP produced a more pronounced apoptotic response than etoposide (ETP) and its liposomal counterpart (LETP), particularly in the A549 model. LipoBP induced the highest proportion of late apoptotic cells (73.13%), while LETP (45.59% late apoptosis) showed a stronger apoptotic response than ETP (38.92% late apoptosis), inducing a mildly increased rate of necrotic cell death (15.11%). Gene-expression analysis supported these findings, as LipoBP upregulated pro-apoptotic genes B*AX, CASP6, CASP3 and CASP9* while downregulating survival-associated genes (BCL-XL, IQSEC2), consistent with activation of the intrinsic apoptotic pathway. Overall, this in vitro study demonstrates the potential of a “liposomal bromophenol” strategy to enhance the anticancer effects of bromophenolic compounds under controlled experimental conditions. Although LipoBP exhibited a more pronounced apoptotic response than etoposide under the studied conditions, such comparisons should be interpreted cautiously due to mechanistic and physicochemical differences between the compounds. In addition, while short-term stability was verified, long-term physical stability—an essential parameter for biomedical translation—remains to be investigated in future studies. Future studies should focus on in vivo pharmacokinetics, tumor-targeted delivery strategies, long-term stability evaluation, and combination therapy approaches to validate these findings in vivo and assess the clinical relevance and translational potential of LipoBP.

## Data Availability

The datasets generated during and/or analyzed during the current study are available from the corresponding author on reasonable request.
